# Supplementary figures and images for: Membrane-mimetic thermal proteome profiling (MM-TPP) toward mapping membrane protein–ligand dynamic interactions
Source: eLife. 2025 Nov 12;14:RP104549. doi: 10.7554/eLife.104549 (PMC12611261; doi:10.7554/eLife.104549)

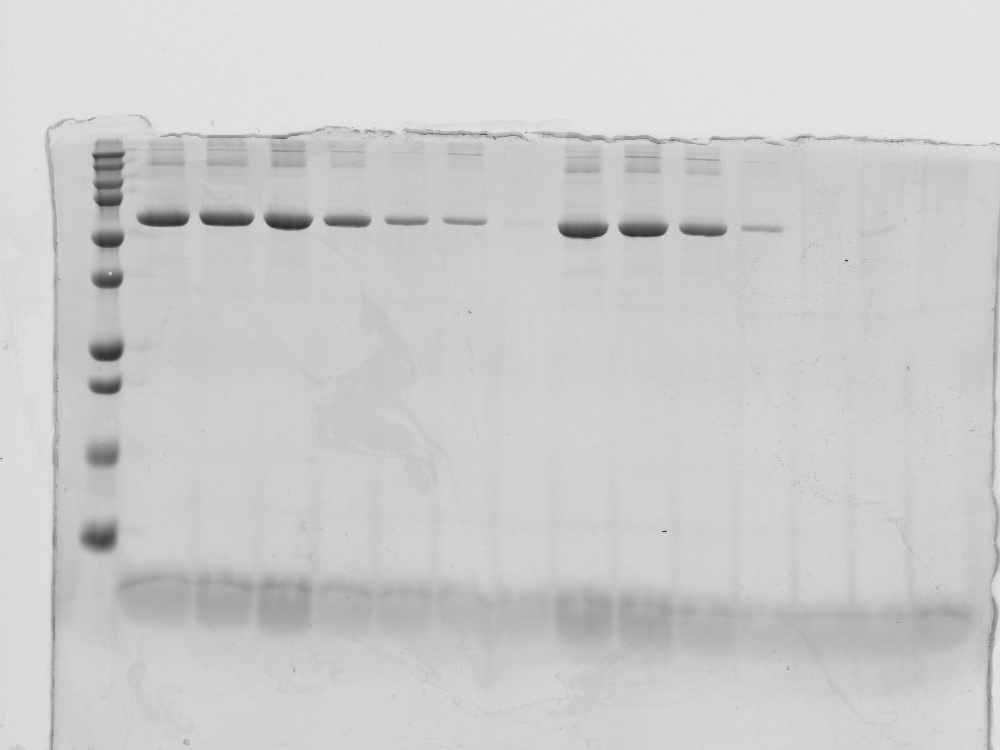

Supplement: Figure 2—source data 1. [file elife-104549-fig2-data1.zip › Figure 2 source data 1/Fig 2A raw gel.tif]

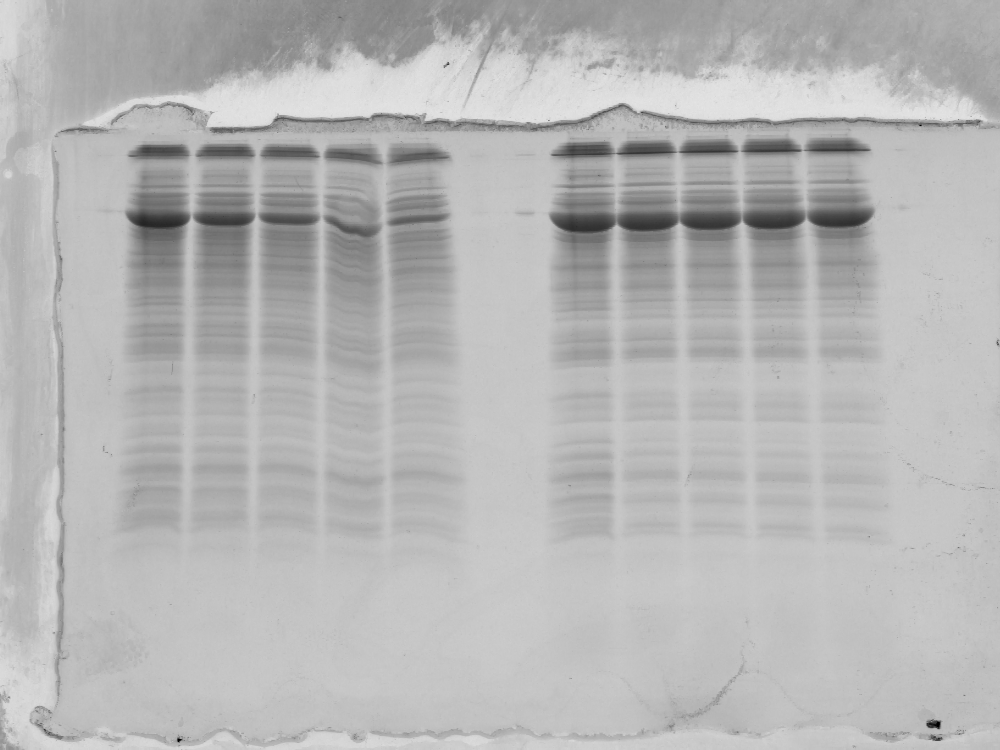

Supplement: Figure 2—source data 1. [file elife-104549-fig2-data1.zip › Figure 2 source data 1/Figure 2 figure supplement 1A raw gel.tif]

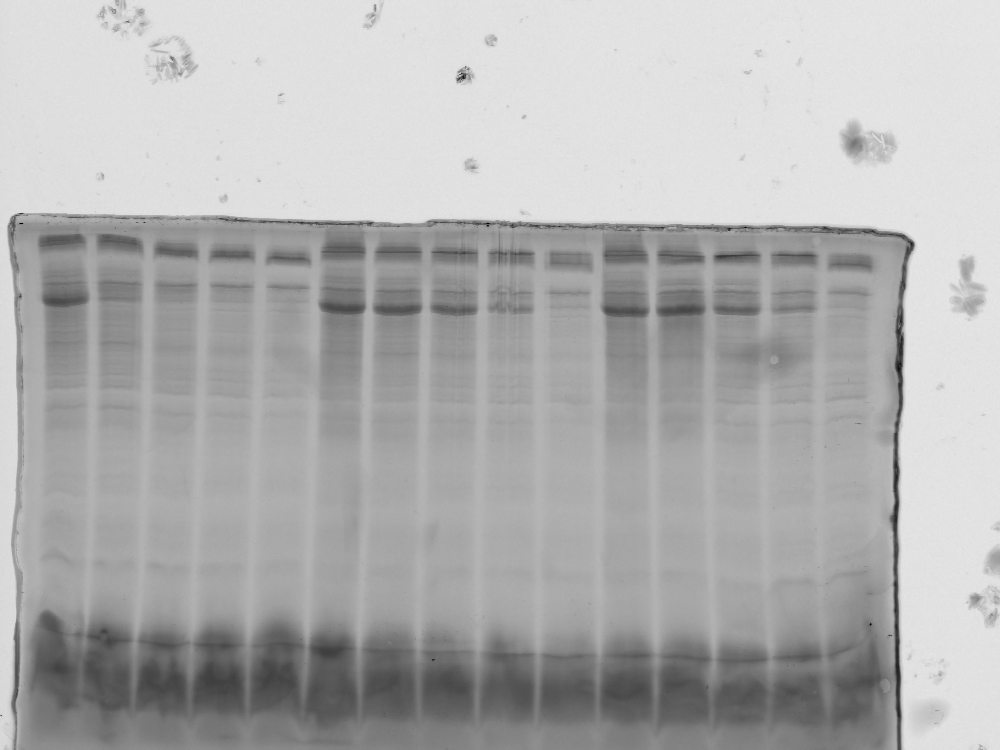

Supplement: Figure 2—source data 1. [file elife-104549-fig2-data1.zip › Figure 2 source data 1/Figure 2 figure supplement 1B raw gel.tif]
