# Supplementary material for: Membrane-mimetic thermal proteome profiling (MM-TPP) toward mapping membrane protein–ligand dynamic interactions
Source: eLife. 2025 Nov 12;14:RP104549. doi: 10.7554/eLife.104549 (PMC12611261; doi:10.7554/eLife.104549)
Supplement: Figure 2—source data 2. [file elife-104549-fig2-data2.zip › Figure 2 source data 2/Figure 2 source data 2.pdf]

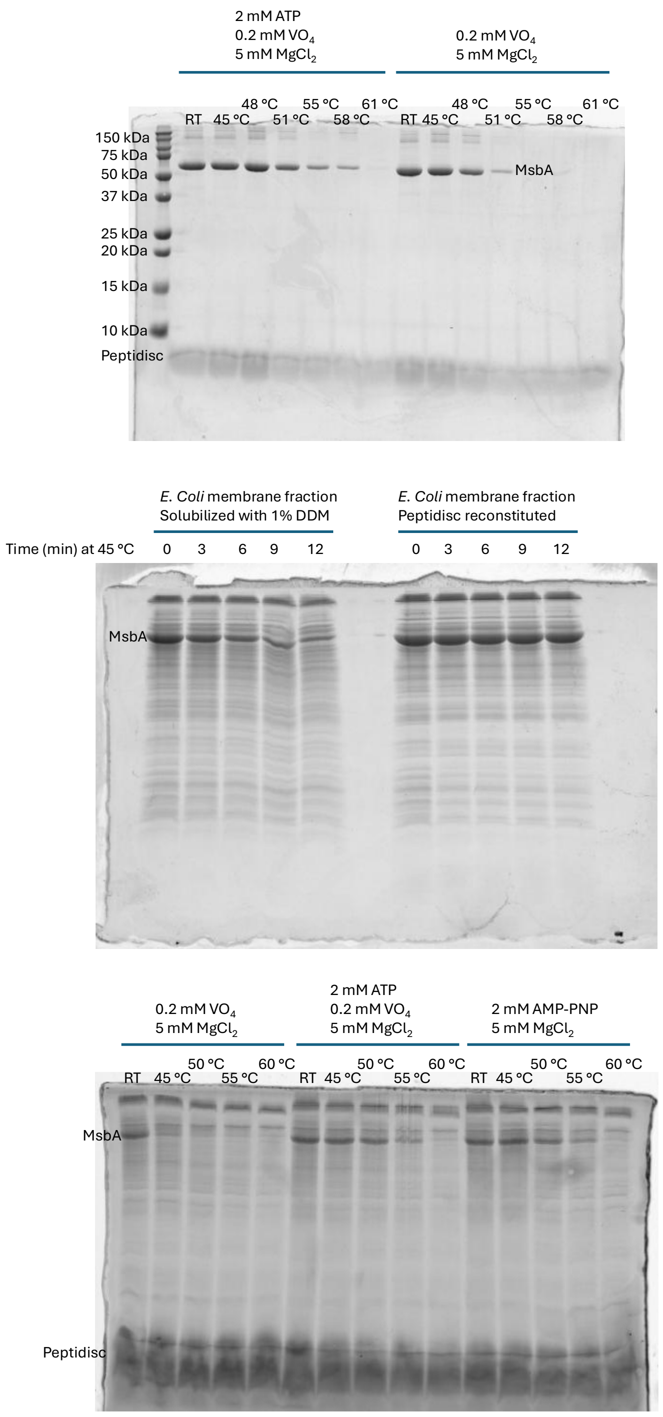

Figure 2, Source Data 2. Original gel images corresponding to Figure 2, panel A, and Figure 2—figure supplement 1, panels A and B. Precision Plus Protein Unstained Standards (Bio-Rad) were used as the molecular weight ladder for Figure 2, panel A. Gels were scanned using an Amersham Typhoon imager with the IR Short channel. For the gels shown in Figure 2, panel A, and Figure 2—figure supplement 1, panel B, samples were incubated at the indicated temperatures for 3 minutes.
