# Supplementary material for: Membrane-mimetic thermal proteome profiling (MM-TPP) toward mapping membrane protein–ligand dynamic interactions
Source: eLife. 2025 Nov 12;14:RP104549. doi: 10.7554/eLife.104549 (PMC12611261; doi:10.7554/eLife.104549)
Supplement: Supplementary file 2. — The % value is used to assess IMP loss relative to total protein count. Each protein was identified based on at least two unique peptides (n=2). [file elife-104549-supp2.docx]

| Temperature | Total protein | Total IMP |
| --- | --- | --- |
| Room temperature | 998 ± 2 | 470 ± 3 (47%) |
| 45 °C | 892 ± 11 | 425 ± 1 (48%) |
| 50 °C | 810 ± 16 | 385 ± 7 (48%) |
| 55 °C | 734 ± 46 | 354 ± 22 (48%) |
| 60 °C | 566 ± 33 | 276 ± 11 (49%) |
| 65 °C | 524 ± 4 | 264 ± 3 (50%) |

**Supplementary File 2:** **Total and Integral Membrane Protein Counts Across Temperatures in Mouse Liver Peptidisc Libraries.** The % value is used to assess IMP loss relative to total protein count. Each protein was identified based on at least two unique peptides (n = 2)
