# Supplementary material for: Membrane-mimetic thermal proteome profiling (MM-TPP) toward mapping membrane protein–ligand dynamic interactions
Source: eLife. 2025 Nov 12;14:RP104549. doi: 10.7554/eLife.104549 (PMC12611261; doi:10.7554/eLife.104549)
Supplement: Supplementary file 3. — Counts are from MaxQuant analyses using a data-dependent acquisition (DDA) workflow across the temperatures tested in membrane-mimetic thermal proteome profiling (MM-TPP) (with ATP–VO4, 2-MeS-ADP, and AMP-PNP) and in detergent-based thermal proteome profiling (DB-TPP) (with ATP–VO4). [file elife-104549-supp3.docx]

| Target | Treatment | Number of unique peptides | | | |
| --- | --- | --- | --- | --- | --- |
|  |  | **51 °C** | **56/57 °C** | **60/61 °C** | **64 °C** |
| MsbA | ATP–VO₄ | Control: 13,12,6 | Control:11,4,9 | Control:7,5,6 |  |
|  |  | Treatment: 16,16,15 | Treatment:17,15,17 | Treatment: 14,11,14 |  |
| ABCA6 | ATP–VO₄ | Control: 9,12,13 | Control:5,3,6 | Control:1,0,3 | Control:1,0,0 |
|  |  | Treatment:21,19,23 | Treatment:12,15,17 | Treatment:12,13,11 | Treatment:7,7,9 |
|  | ATP–VO₄ (DB-TPP) | Control:22,25,25 | Control:25,27,23 | Control:22,23,22 | Control:14,19,15 |
|  |  | Treatment:25,25,24 | Treatment:22,24,23 | Treatment:17,5,22 | Treatment:13,20,15 |
|  | 2-MeS-ADP | Control:6,5,5 | Control:0,1,0 |  | |
|  |  | Treatment:1,5,7 | Treatment:2,2,1 |  |  |
|  | AMP-PNP | Control:6,5,5 | Control:3,1,5 | Control:1,2,3 | Control:0,0,0 |
|  |  | Treatment:6,7,6 | Treatment:3,6,4 | Treatment:2,0,1 | Treatment:0,0,0 |
| ABCB11 | ATP–VO₄ | Control:13,10,15 | Control:0,3,4 | Control:1,0,0 | Control:0,1,0 |
|  |  | Treatment:21,22,21 | Treatment:6,13,12 | Treatment:6,10,9 | Treatment:1,2,3 |
|  | ATP–VO₄ (DB-TPP) | Control:30,32,29 | Control:26,25,27 | Control:22,20,24 | Control:16,15,16 |
|  |  | Treatment:38,37,38 | Treatment:30,32,23 | Treatment:20,5,19 | Treatment:15,23,17 |
|  | 2-MeS-ADP | Control:0,0,0 | Control:0,0,0 |  | |
|  |  | Treatment:0,0,0 | Treatment:0,0,0 |  |  |
|  | AMP-PNP | Control:6,6,5 | Control:1,2,3 | Control:1,2,1 | Control:0,0,0 |
|  |  | Treatment:8,8,7 | Treatment:7,3,4 | Treatment:2,0,4 | Treatment:0,0,0 |
| ABCB6 | ATP–VO₄ | Control:0,1,1 | Control:0,0,0 | Control:0,0,0 | Control:0,0,0 |
|  |  | Treatment:3,3,3 | Treatment:0,0,0 | Treatment:0,0,0 | Treatment:0,0,0 |
|  | ATP–VO₄ (DB-TPP) | Control:0,0,0 | Control:0,0,0 | Control:0,0,0 | Control:0,0,0 |
|  |  | Treatment:0,0,0 | Treatment:0,0,0 | Treatment: | Treatment:0,0,0 |
|  | 2-MeS-ADP | Control:0,0,0 | Control:0,0,0 |  | |
|  |  | Treatment:0,0,0 | Treatment:0,0,0 |  |  |
|  | AMP-PNP | Control:0,0,0 | Control:0,0,0 | Control:0,0,0 | Control:0,0,0 |
|  |  | Treatment:3,2,2 | Treatment:2,2,2 | Treatment:0,0,0 | Treatment:0,0,0 |
| ABCC2 | ATP–VO₄ | Control:6,4,9 | Control:0,0,0 | Control:0,0,0 | Control:0,0,0 |
|  |  | Treatment:13,15,10 | Treatment:6,6,5 | Treatment:3,5,2 | Treatment:0,0,0 |
|  | ATP–VO₄ (DB-TPP) | Control:13,15,15 | Control:16,11,12 | Control:8,10,8 | Control:3,3,5 |
|  |  | Treatment:22,19,16 | Treatment:17,16,15 | Treatment:7,6,11 | Treatment:8,12,6 |
|  | 2-MeS-ADP | Control:2,2,4 | Control:0,0,0 |  | |
|  |  | Treatment:3,1,6 | Treatment:1,2,1 |  |  |
|  | AMP-PNP | Control:1,1,3 | Control:0,0,0 | Control:0,0,0 | Control:0,0,0 |
|  |  | Treatment:4,3,2 | Treatment:0,0,0 | Treatment:0,0,0 | Treatment:0,0,0 |
| ABCC3 | ATP–VO₄ | Control:10,6,7 | Control:0,0,0 | Control:0,0,0 | Control:0,0,0 |
|  |  | Treatment:13,15,15 | Treatment:0,0,0 | Treatment:2,3,5 | Treatment:0,0,0 |
|  | ATP–VO₄ (DB-TPP) | Control:0,0,0 | Control:0,0,0 | Control:0,0,0 | Control:0,0,0 |
|  |  | Treatment:0,0,0 | Treatment:0,0,0 | Treatment:0,0,0 | Treatment:0,0,0 |
|  | 2-MeS-ADP | Control:0,0,0 | Control:0,0,0 |  | |
|  |  | Treatment:0,0,0 | Treatment:0,0,0 |  |  |
|  | AMP-PNP | Control:0,0,0 | Control:0,0,0, | Control:0,0,0 | Control:0,0,0 |
|  |  | Treatment:0,0,0 | Treatment:0,0,0 | Treatment:0,0,0 | Treatment:0,0,0 |
| ABCC9 | ATP–VO₄ | Control:15,14,13 | Control:6,5,6 | Control:2,0,4 | Control:1,0,2 |
|  |  | Treatment:15,16,17 | Treatment:5,8,9 | Treatment:7,13,10 | Treatment:8,7,6 |
|  | ATP–VO₄ (DB-TPP) | Control:1,1,1 | Control:1,1,1 | Control:1,1,1 | Control:1,1,1 |
|  |  | Treatment:1,1,1 | Treatment:1,1,1 | Treatment:1,1,1 | Treatment:1,1,1 |
|  | 2-MeS-ADP | Control:16,19,18 | Control:6,8,3 |  | |
|  |  | Treatment:15,18,20 | Treatment:3,12,8 |  |  |
|  | AMP-PNP | Control:13,12,11 | Control:5,7,7 | Control:5,9,6 | Control:4,3,6 |
|  |  | Treatment:13,13,13 | Treatment:11,9,9 | Treatment:7,3,8 | Treatment:3,5,4 |
| ABCG2 | ATP–VO₄ | Control:6,8,9 | Control:6,6,5 | Control:2,0,1 | Control:0,0,1 |
|  |  | Treatment:8,7,9 | Treatment:8,8,7 | Treatment:9,8,7 | Treatment:4,5,5 |
|  | ATP–VO₄ (DB-TPP) | Control:10,11,11 | Control:9,9,10 | Control:5,7,6 | Control:4,4,4 |
|  |  | Treatment:10,11,12 | Treatment:10,10,7 | Treatment:3,1,5 | Treatment:2,6,4 |
|  | 2-MeS-ADP | Control:4,3,3 | Control:1,1,0 |  | |
|  |  | Treatment:3,3,3 | Treatment:0,1,0 |  |  |
|  | AMP-PNP | Control:3,3,3 | Control:1,0,0 | Control:0,0,0 | Control:0,0,0 |
|  |  | Treatment:3,3,3 | Treatment:3,3,3 | Treatment:3,2,3 | Treatment:0,0,0 |
| ABCG5 | ATP–VO₄ | Control:1,1,1 | Control:0,0,0 | Control:0,0,0 | Control:0,0,0 |
|  |  | Treatment:2,2,2 | Treatment:0,0,0 | Treatment:0,0,0 | Treatment:0,0,0 |
|  | ATP–VO₄ (DB-TPP) | Control:2,1,2 | Control:1,1,1 | Control:0,0,0 | Control:0,0,0 |
|  |  | Treatment:2,2,1 | Treatment:1,0,1 | Treatment:0,0,0 | Treatment:0,0,0 |
|  | 2-MeS-ADP | Control:0,0,0 | Control:0,0,0 |  | |
|  |  | Treatment:0,0,0 | Treatment:0,0,0 |  |  |
|  | AMP-PNP | Control:0,0,0 | Control:0,0,0 | Control:0,0,0 | Control:0,0,0 |
|  |  | Treatment:0,0,0 | Treatment:0,0,0 | Treatment:0,0,0 | Treatment:0,0,0 |
| BCS1l | ATP–VO₄ | Control: 0,0,0 | Control:0,0,0 | Control: 0,0,0 | Control: 0,0,0 |
|  |  | Treatment: 6,7,6 | Treatment: 1,4,2 | Treatment:0,0,0 | Treatment: 1,0,0 |
|  | ATP–VO₄ (DB-TPP) | Control: 1,4,4 | Control:1,0,1 | Control: 0,0,0 | Control: 0,0,0 |
|  |  | Treatment: 7,5,4 | Treatment: 0,2,3 | Treatment: 0,0,0 | Treatment: 1,0,0 |
|  | 2-MeS-ADP | Control: 0,0,0 | Control: 0,0,0 |  |  |
|  |  | Treatment:,0,0,0 | Treatment: 0,0,0 |  |  |
|  | AMP-PNP | Control: 0,0,1 | Control:0,0,0 | Control:0,0,0 | Control:0,0,0 |
|  |  | Treatment:13.10.12 | Treatment:15,15,15 | Treatment:15,4,12 | Treatment:16,13,9 |
| P2RX4 | ATP–VO₄ | Control: 3, 2, 3 | Control: 3,3,2 | Control: 0,0,0 | Control:3,4,5 |
|  |  | Treatment: 3,3,1 | Treatment:1,0,1 | Treatment: 0,0,0 | Treatment:0,1,3 |
|  | ATP–VO₄ (DB-TPP) | Control: 3,1,4 | Control: 2,3,2 | Control: 4,3,3 | Control:1,1,2 |
|  |  | Treatment:2,1,1 | Treatment: 1,1,2 | Treatment:1,2,2 | Treatment: 1,2,2 |
|  | 2-MeS-ADP | Control: 0,0,0 | Control: 0,0,0 |  |  |
|  |  | Treatment: 0,0,0 | Treatment: 0,0,0 |  |  |
|  | AMP-PNP | Control:2,2,2 | Control:2,2,2 | Control:2,2,2 | Control:2,2,2 |
|  |  | Treatment:2,1,2 | Treatment:2,1,1 | Treatment:2,0,2 | Treatment:2,2,2 |
| P2RY6 | ATP–VO₄ | Control: 12, 13, 13 | Control:2.2.3 | Control:1,0,0 | Control:0,0,0 |
|  |  | Treatment:12,10,13 | Treatment:3,5,6 | Treatment:2,4,3 | Treatment:0,0,0 |
|  | ATP–VO₄ (DB-TPP) | Control: 22,22,22 | Control: 4,2,3 | Control:0,0,1 | Control: 0,1,0 |
|  |  | Treatment: 25,23,25 | Treatment: 3,1,5 | Treatment: 0,0,1 | Treatment: 1,0,0 |
|  | 2-MeS-ADP | Control: 2,2,2 | Control: 1,1,0 |  |  |
|  |  | Treatment: 0,2,2 | Treatment: 1,1,1 |  |  |
|  | AMP-PNP | Control:7,5,6 | Control:1,0,0 | Control:0,0,0 | Control: 0,0,0 |
|  |  | Treatment:8,8,4 | Treatment:1,2,3 | Treatment: 0,0,0 | Treatment: 0,0,0 |
| P2RY12 | ATP–VO₄ | Control:19,15,15 | Control:2,5,4 | Control:0,0,1 | Control:0,0,0 |
|  |  | Treatment:14,14,13 | Treatment:3,6,9 | Treatment: 8,9,8 | Treatment: 5,4,5 |
|  | ATP–VO₄ (DB-TPP) | Control:29, 30, 30 | Control: 22, 21, 25 | Control: 20, 16, 18 | Control:14, 12, 14 |
|  |  | Treatment:29, 28, 29 | Treatment: 23, 21, 23 | Treatment: 19,11,17 | Treatment:13,16,14 |
|  | 2-MeS-ADP | Control: 6,4,7 | Control: 2,3,4 |  | |
|  |  | Treatment: 7,6,7 | Treatment: 4,3,6 |  |  |
|  | AMP-PNP | Control:2,1,2 | Control:0,0,0 | Control:0,0,0 | Control:0,0,0 |
|  |  | Treatment:3,2,2 | Treatment:0,0,0 | Treatment:0,0,0 | Treatment:0,0,0 |
| Mao-B | ATP–VO₄ | Control:20,18,18 | Control:15,14,14 | Control:14,0,13 | Control:1,1,1 |
|  |  | Treatment:20,21,20 | Treatment:16,14,17 | Treatment:14,17,18 | Treatment:14,15,15 |
|  | ATP–VO₄ (DB-TPP) | Control:25,24,26 | Control:25,24,27 | Control:21,19,21 | Control:15,15,18 |
|  |  | Treatment:26,23,26 | Treatment:25,21,21 | Treatment:17,6,19 | Treatment:14,17,18 |
|  | 2-MeS-ADP | Control:23,21,22 | Control:20,17,17 |  | |
|  |  | Treatment:18,24,24 | Treatment:20,18,19 |  |  |
|  | AMP-PNP | Control:15,17,16 | Control:10,13,13 | Control:13,13,14 | Control:8,9,8 |
|  |  | Treatment:17,17,17 | Treatment:15,12,13 | Treatment:10,10,14 | Treatment:10,9,7 |

**Supplementary File 3:** **Unique peptide counts for proteins highlighted in this study.**

MsbA, ABCA6, ABCB1, ABCB6, ABCC2, ABCC3, ABCC9, ABCG2, ABCG5, BCS1L, P2RX4, P2RY6, P2RY12, and MAO-B. Counts are from MaxQuant analyses using a data-dependent acquisition (DDA) workflow across the temperatures tested in MM-TPP (with ATP–VO₄, 2-MeS-ADP, and AMP-PNP) and in DB-TPP (with ATP–VO₄).
